# Statistical algorithms for ontology-based annotation of scientific literature

**DOI:** 10.1186/2041-1480-5-S1-S2

**Published:** 2014-06-03

**Authors:** Chayan Chakrabarti, Thomas B Jones, George F Luger, Jiawei F Xu, Matthew D Turner, Angela R Laird, Jessica A Turner

**Affiliations:** 1 Department of Computer Science, University of New Mexico, Albuquerque, New Mexico, USA; 2 Mind Research Network, Albuquerque, New Mexico, USA; 3 Department of Physics, Florida International University, Miami, Florida, USA; 4 Department of Psychology and the Neuroscience Institute, Georgia State University, Atlanta, Georgia, USA

## Abstract

**Background:**

Ontologies encode relationships within a domain in robust data structures that can be used to annotate data objects, including scientific papers, in ways that ease tasks such as search and meta-analysis. However, the annotation process requires significant time and effort when performed by humans. Text mining algorithms can facilitate this process, but they render an analysis mainly based upon keyword, synonym and semantic matching. They do not leverage information embedded in an ontology's structure.

**Methods:**

We present a probabilistic framework that facilitates the automatic annotation of literature by indirectly modeling the restrictions among the different classes in the ontology. Our research focuses on annotating human functional neuroimaging literature within the Cognitive Paradigm Ontology (CogPO). We use an approach that combines the stochastic simplicity of naïve Bayes with the formal transparency of decision trees. Our data structure is easily modifiable to reflect changing domain knowledge.

**Results:**

We compare our results across naïve Bayes, Bayesian Decision Trees, and Constrained Decision Tree classifiers that keep a human expert in the loop, in terms of the quality measure of the F1-mirco score.

**Conclusions:**

Unlike traditional text mining algorithms, our framework can model the knowledge encoded by the dependencies in an ontology, albeit indirectly. We successfully exploit the fact that CogPO has explicitly stated restrictions, and implicit dependencies in the form of patterns in the expert curated annotations.

## Background

Advances in neuroimaging and brain mapping have generated a vast amount of scientific knowledge. This data, gleaned from a large number of experiments and studies, pertains to the functions of the human brain. Given large bodies of properly annotated research papers, it is possible for researchers to use meta-analysis tools to identify and understand consistent patterns in the literature. Since researchers often use jargon which is specific to a small sub-field to describe their experiments, it is helpful to tag papers with standardized descriptions of the experimental conditions of each paper's accompanying study. Several repositories have been created with this effort in mind.

BrainMap (http://www.brainmap.org) is one of the largest and most widely used repositories of neuroimaging results. The BrainMap software suite provides computational toolsets, scientific data sets, and other informatics resources needed to explore the different cognitive constructs underlying brain function in various disorders, such as the constellation of schizophrenia, bipolar disorder, depression, and autism [1]. Large-scale quantitative meta-analyses demand the ability to easily identify studies using the same (or similar enough) experimental methods and subjects. The BrainMap method for describing experiments has evolved into a taxonomy composed chiefly of structured keywords that categorize the experimental question addressed, the imaging methods used, the behavioral conditions during which imaging was acquired, and the statistical contrasts performed.

The Cognitive Paradigm Ontology (CogPO), compliant with the Basic Formal Ontology (BFO) [2], builds upon the BrainMap repository on the understanding that while the experimental psychology and cognitive neuroscience literature may refer to certain behavioral tasks by name (e.g., the Stroop task or the Sternberg paradigm) or by function (a working memory task, a visual attention task), the presentation of these paradigms in the literature can vary tremendously and are most precisely characterized by the unique combination of the stimuli that are presented to the subject, the response expected from the subject, and the instructions given to the subject. The prevalent use of different terminologies for the same paradigm across different sub-specialities can hinder assimilation of coherent scientific knowledge. Discovering equivalence among these terminologies in a structured coherent fashion will facilitate richer information retrieval operations. The BrainMap repository structure forms the backbone of the Cognitive Paradigm Ontology. It includes the keywords from BrainMap, as well as others, and explicitly represents the implicit definitions and relationships among them [2]. This allows published experiments implementing similar behavioral task characteristics to be linked, despite the use of alternate vocabularies.

Each piece of literature from the BrainMap repository is annotated according to the CogPO definitions. The process of annotation is traditionally undertaken by a human subject matter expert, who decides the suitable annotation terms from the CogPO schema after reading the paper, while extracting descriptions of first PET and then fMRI experiments, and storing each paper's results in a standardized system for ease of retrieval [2,3]. Unfortunately, this task is both time and effort intensive. It presents a major bottleneck and cost to the whole process. As a result, even though the value of the BrainMap project has been proven, the number of publications in the literature far outweighs the number of publications that have been included in the database [3]. In this study, we propose solutions for replacing this human only annotation step with automated suggestions for the experimental paradigm terms.

### Text mining

Text mining methods have found application in identifying patterns and trends in rich textual data [4-6]. Text mining algorithms have also been extended to the problem of multi-objective multi-label classification where a variety of predictive functions can be constructed depended on the required objective function including optimizing an F1-score [7] or minimizing the hamming loss [8]. F1 score is the geometric mean of the recall, a measure of the classifier's tendency to return all of the correct labels, and accuracy, a measure of the tendency of labels returned by the classifier to be correct. Hamming loss, on the other hand, gives a count of the number of false positives and false negatives a classifier identifies. Both of these distinct measures give an indication of the classifier's ability to return high quality classifications.

The performance of multi-objective multi-label classification can be further optimized using regret analysis [9]. The binary relevance method has been used to extend the solution of multi-objective multi-label classification methods to larger datasets [10]. The main algorithms for multi-objective multi-label classification are generally classified under the umbrellas of problem transformation, algorithm adaptation, lazy learning, support vector machine derived, ensemble methods, and label dependence exploitation [11]. Support Vector Machines and Self Organizing Feature Maps have been used to reduce the inherently high dimensionality of text mining problems [12] and have shown promising results [13]. Other, perception based techniques, like artificial neural networks and radial basis functions are useful in estimating classification functions for classes of problems with non-linear and irregular decision boundaries [14].

Latent Semantic Analysis works on the assumption that words that are close in meaning occur close to each other in a document [15,16]. Using Singular Value Decomposition, the matrix representing word counts by paragraph from large document clusters are reduced to only preserve the similarity metric among documents. Documents can then be compared using projections and other distance metrics. K-means clustering partitions a corpus of documents in to clusters, where each cluster refers to similar documents [17]. There are many variations on this theme. In fuzzy co-means clustering, each document may belong to more than one cluster defined by a fuzzy function [18,19]. Similarly, a variant of the classic Expectation-Maximization algorithm assigns probabilistic distribution function among the clusters to each document [20].

The NCBO Annotator takes free text and uses efficient concept-recognition techniques to suggest annotations from the Bio-Portal repository of ontologies [21]. The Neuroscience Information Framework [22] uses ontological annotations of a broad variety of neuroscience resources to retrieve information for user queries.

However, most text-mining techniques do not leverage the hierarchical structures encoded implicitly in an ontology. They consider the ontology terms as anchors for clustering or topic modeling techniques, but have no way to use the information that the terms may have exploitable relations to each other, either causal or hierarchical. These terms could just be a set of high entropy keywords for the algorithms to be equally effective. We present a framework that makes use of some of the hierarchical information that is available from the ontology itself for the annotation task.

Ontology-based annotation of documents has been an important application area for text mining research [23]. Since the interdisciplinary nature of this text mining applied to ontologies leads to overlap of terminology for both fields, we clarify the terms we use here. We use *categories *to denote specific superclasses in CogPO (e.g., "Stimulus Type"), and *labels *to denote the leaf terms in each class, which are actually applied to the abstracts (e.g., "Flashing Checkerboard", which is a subclass of "Stimulus Type"). *Dependencies *refer to the explicit *interaction *between the *ontology *and the *specific corpora*, as captured by the expert-assigned *annotations*. This is an implicit function of the *interrelationships *between *classes *(categories of labels)*, leaf *terms, the inherent (but not explicitly stated) logical *restrictions *in CogPO, and the manner in which those *relationships *are reified in a specific corpus by human annotators.

In previous work using a similar dataset, we evaluated a version of k-nearest-neighbor (kNN) for performing automated annotations [24,25]. We found that the performance was comparable with results on other textual annotation datasets, but fairly poor for the multi-label aspects of the problem. Text mining algorithms have also been applied to the problem of multi-label annotation; the general case in which there are more than two labels to choose from, and each paper can be best described by more than one label [8,26].

## Methods

We demonstrate techniques for automatic annotation of the neuroimaging literature driven by the Cognitive Paradigm Ontology.

### Corpus

**Figure 1:**
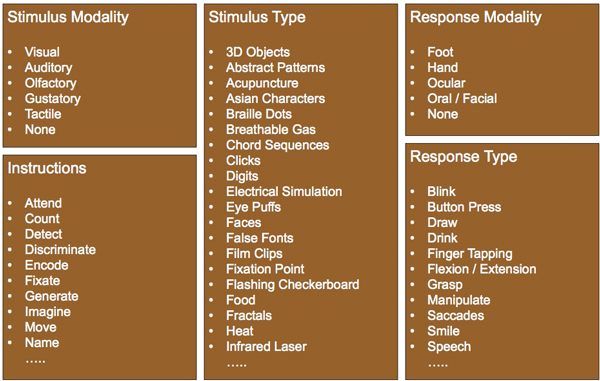
**CogPO annotations**. We consider annotations from 5 distinct categories: Stimulus Modality, Stimulus Type, Response Modality, Response Type, and Instructions. A subset of the labels for each category is shown here.

Our corpus consists of 247 human subject matter expert annotated abstracts that are part of the BrainMap database. We consider annotations in 5 distinct categories for each abstract - *Stimulus Modality *(SM), *Stimulus Type *(ST), *Response Modality *(RM), *Response Type *(RT) and *Instructions *(I). Each of these categories is comprised of several labels as described in CogPO (Turner & Laird 2012) as shown in Figure 1. These human subject matter expert annotated abstracts serve as the gold standard against which we test our stochastic approaches. Table 1 shows a component of the schema from CogPO that we consider along with a subset of the labels. We only work on the abstracts, and not the full paper, because we want to interface our tool directly with the eUtils toolkit of PubMed that can retrieve the text of abstracts in batch [27].

**Table 1:** Overview of key terms from the CogPO Ontology (adapted from [1]).

Concepts	Parent Class	Definition
Stimulus Role	BFO: role	The role of a stimulus in a behavioral experiment is attributed to the object(s) that are presented to the subject in a controlled manner in the context of the experiment.

Response Role	BFO: role	The role of response is attributed to the overt or covert behavior that is elicited from the subject in an experimental condition.

Stimulus	BFO: ObjectAggregate	The object or set of objects, internal or external to the subject, which is intended to generate either an overt or covert response in the subject as part of an experimental condition.

Response	BFO: ProcessAggregate	The overt or covert behavior that is elicited from the subject in an experimental condition.

Instructions	IAO:'action specification', BFO: generically_ independent_continuant	Instructions are the information-bearing entity that sets up the rules for desired behavior from the subjects. An explicit direction that guides the behavior of the subject during the experimental conditions. Instructions serve the function that they lay out what the response behaviors should be for any set of stimuli in the experiment.

Stimulus Modality	BFO: Quality	The quality of the sensory perception of an explicit stimulus.

Response Modality	BFO: FiatObjectPart	Class of body parts used to perform the actions which can play the role of an overt response

We consider only a subset of the Cognitive Paradigm Ontology as defined in [1]. We consider 5 classes, *Stimulus Modality, Stimulus Type, Response Modality, Response Type*, and *Instructions*.

Each abstract is annotated by at least one label from each of the SM, ST, RM, RT, or I categories, and possibly multiple labels from each. The average number of labels per category per abstract ranged from 1.15 to 1.85 depending on the category. The human curator's annotations model implicit dependencies as a result of the CogPO-corpus interaction. These dependencies will be specific for each different corpus of abstracts.

The CogPO ontology explicitly includes restrictions on the labels, e.g., a *Tone *as a *Stimulus Type *label entails that the *Stimulus Modality *must include *Auditory*, or the *Instruction *label *Smile *entails *Facial *as the label for *Response Modality*. A flat text mining approach would be unable to make these distinctions, i.e., it would not be able to tell that label *a *can change the probability of label *b*, in some other category. Our approach indirectly models this by learning patterns from the expert curated corpus.

### Naïve Bayes

Naïve Bayes is a probabilistic learning method, based on Bayes' rule, which works surprisingly well on problems where a strong independence hypothesis assumption is not met. In fact, naïve Bayes also works well for supervised learning when the number of instances in the training set is relatively small, which is our situation [25]. It has been extended to the multi-label scenario using various transformation techniques [9]; we have also found in a comparison of text mining methods applied to this corpus that a naïve Bayes approach works better than several others [25]. Therefore, we start with a naïve Bayes approach.

The naïve Bayes technique across all categories and possible labels does not leverage the dependencies between labels in different categories, which are implicitly encoded in the domain ontology. Traditional text-mining techniques consider the labels to be anchors for clustering or topic modeling techniques, but have no way to use the fact that the anchors may have implicit dependencies to each other and to object features. The features used to derive terms in traditional text mining are often a set of high entropy keywords [5]. Our framework does not explicitly model the interrelationships and restrictions in CogPO, but we exploit the fact that these relations and restrictions do exist and implicitly model the information that is encoded in the ontology. This is an important distinguishing characteristic of our stochastic approach.

In many ontologies, there are often different classes from which a label may be drawn [1]. While naïve Bayes is able to assign certain features in a training sample to labels in a single category, it is unable to learn about dependencies between labels and their associated attributes in different categories. Further, it is not possible for naïve Bayes alone to increase or decrease its confidence in one label after it has been informed that some other label is a correct or incorrect annotation for the same sample. Our method expands on naive Bayes by restricting training sets at each node in the tree to only those training objects pertinent to that node. This allows us to take advantage of any underlying dependencies in the training set between labels of different categories, which would otherwise be hidden by building a separate classifier for each category.

### Formal framework of naïve Bayes

**Figure 2:**
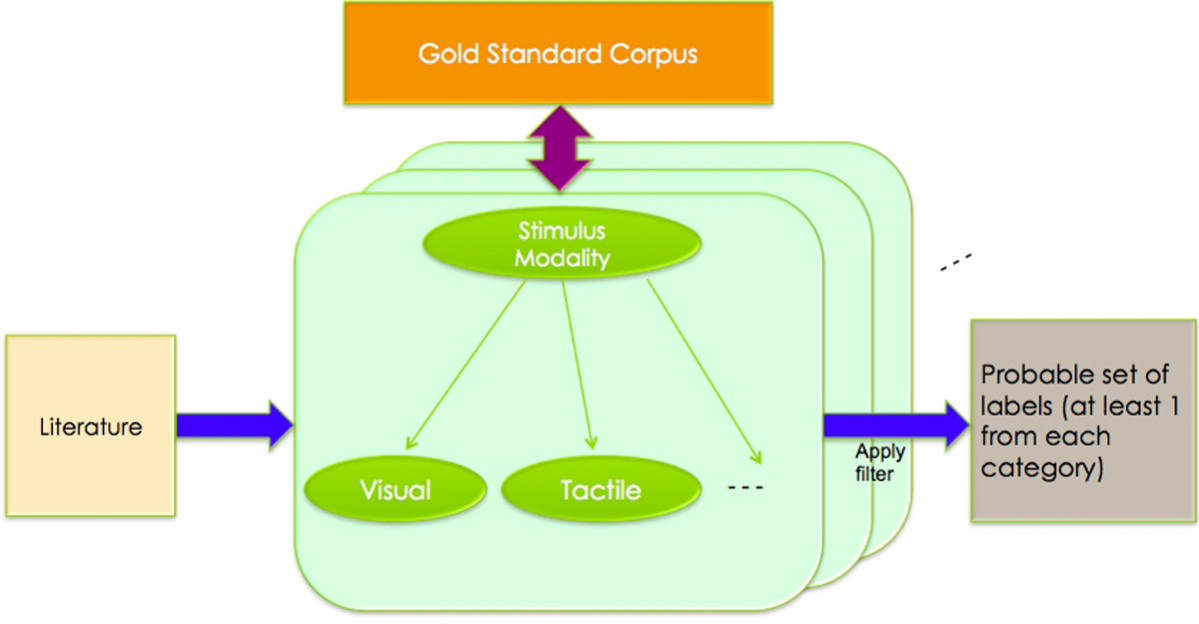
**Naïve Bayes**. Naïve Bayes determines most probable labels in a category.

The framework which Naive Bayes requires to operate includes a set of items to be classified whose classifications have already been obtained through some other process (usually a human annotator). Each item in this study, abstracts, which have been tagged with labels from the CogPo ontology, is then recast as a feature vector. In our work, this feature vector is a Boolean vector with one bit for every non-stop word in the corpus. Each bit in an abstract's associated feature vector is set to true if the word occurs in the abstract and false otherwise. Figure 2. shows an overview of the naïve Bayes method.

More formally, we define the set of abstracts, the feature vector, and the set of feature vectors (representing words from the corpus that are not stop words) as follows.

**Definition 1**. The set of abstracts in the corpus is defined as

$$D=\left\{d\;|d\;is\;an\;abstract\;in\;the\;corpus\right\}$$

**Definition 2**. A feature is defined as

F=<f|fisafeaturerepresentinganon-stopword>

**Definition 3**. A feature vector is defined as

$$V=\left\{v_{c}|v_{c}=<b_{c1}\;\ldots \;b_{cn}>,b_{cj}=\left\{\begin{array}{c} TRUE,\;\;\;f_{i}\in d_{c}\\ FALSE,\;\;otherwise \end{array}\right)\right\}$$

By the previous definitions, the length or size of

$$\left|v_{c}\right|=\left|f\right|\;and\;\left|V\right|=\left|D\right|=number\;of\;abstracts$$

**Definition 4**. CogPO itself, as used in this study can be defined as the set of categories *Stimulus Modality, Stiumulus Type, Response Modality, Response Type*, and *Instruction*.

$$C=\left\{SM,ST,RM,RT,I\right\}$$

**Definition 5**. Each category can be defined as a set of labels *l_i. _*So for example,

$$\text{SM}=\left\{l_{1},l_{2},\ldots \right\}$$

with *l_i _= Visual, l_2 _= Auditory*, etc

The other 4 categories, *ST, RM, RT*, and *I*, can be similarly defined.

Now we can explain the mechanism by which naive Bayes classifies each abstract. First, the classifier estimates

$$P\left(M\left(d_{c},l_{j}\right)|b_{ci}=TRUE\right)$$

or the probability that abstract *c *has label *j *given bit i in its feature vector is *TRUE*, by examining the gold standard corpus, extracting only those abstracts which have bit *i *set to *TRUE*, and counting the frequency with which label *j *occurs in this set. This is done for each label and for each of the feature bits. The classifier also estimates

$$P\left(M\left(d_{c},l_{j}\right)|b_{ci}=FALSE\right))$$

for each label and feature by a similar process.

Next the classifier estimates

$$P\left(b_{ci}=TRUE|M\left(d_{c},l_{j}\right)\right)$$

the probability that bit *i *in the feature vector of abstract *x *is true given that abstract *x *is labeled with label *j*, by flipping the above process around and examining only those abstracts which have label *l_j _*and counting the frequency with which *b_cj _*is set to *TRUE *in the annotated corpus. Similarly, the classifier then does this for the cases when *b_ci _*is set to *FALSE*.

Additionally, the classifier estimates

$$P\left(b_{ci}=TRUE\right)$$

by simply looking at the frequency with which the *i^th ^*bit of each abstract's feature vectors is true in the gold standard corpus. Similarly the classifier finds

$$P\left(b_{ci}=FALSE\right)=1-P\left(b_{ci}=TRUE\right)$$

Lastly,

$$P\left(M\left(d_{c},l_{j}\right)\right)$$

the probability that abstract *c *has label *j*, is estimated by counting the frequency of the occurrence of label *j *in the gold standard corpus.

Given these four sets of values

$$P\left(M\left(d_{c},l_{j}\right)|b_{ci}\right),P\left(b_{ci}|M\left(d_{c},l_{j}\right)\right),P\left(b_{ci}\right)$$

and

$$P\left(M\left(d_{c},l_{j}\right)\right)$$

for each label and each feature bit we can estimate

$$P\left(M\left(d_{c}|l_{j}\right)|V_{c}\right)$$

or the probability that an abstract *c *is labeled with label *j *given its feature vector.

Since, for any random variable *A *and *B*,

$$P\left(A|B\right)=P\left(A\wedge B\right)/P\left(B\right)$$

we know that

$$P\left(M\left(d|l_{j}\right)|V,)\right)=P(M(d_{c}|l_{j}\;and\;V_{c})/P\left(V_{c}\right).$$

The *naive *in naive Bayes comes from assuming that the probability of each bit being true in the feature vector is independent of the state of every other bit in the feature vector. Therefore:

$$\begin{array}{c} P\left(M\left(d|l_{j}\right)|V\right)=P\left(M\left(d_{c},l_{j}\right)\wedge V_{c}\right)/P\left(V_{c}\right)\\ \;\approx P\left(M\left(d_{c},l_{j}\right)\right)*\Pi \;I=1\;to\left|F\right|\;P(b_{ci}|M\left(d_{c},l_{j}\right)/P\left(b_{ci}\right) \end{array}$$

Similarly, we calculate the probability for all the other labels in *SM *as well as *ST, RM, RT*, and *I*. We used binary relevance in a single category to solve the multi label classification problem. Our method takes the raw probability calculated by the Bayesian classifier using the above equations for each label and accepts all labels that receive a probability greater than an arbitrary pre-defined cutoff *α*.

Bayesian decision trees

Decision trees are discrete models that can predict the output labels of samples in a data set, based on several input variables arranged in a tree-like structure with nodes and branches. Nodes in the tree represent a decision variable and the branches correspond to the next decision variable to be queried based on the outcome of the previous decision variable. We use the Bayesian classifiers to make decisions about which labels to include at each node while traversing down the tree.

**Definition 6**. *B_C,S _*is a Bayesian classifier trained on set *S ⊆D *over category *C*.

**Definition 7**. If *S *is a training set and *s ∈ S *then *label(s) *is the set of correct labels attached to item s.

**Definition 8**. If *t *is a node in a tree *T *such that each node in *T *contains a label or an empty label, then *L_t∗ _*is a set that contains the label of node *t *and all of the labels of each ancestor of *t*, with no addition made if the label of a node is empty. In practice, the root is the only node that will have an empty label, since on the root node, the naiveBayes algorithm will consider the entire training set.

**Definition 9**. *T *is a Bayesian Decision Tree if each node *t *of *T *consists of a category *C_t _*which is not the same category as any of the ancestors of *t*, and which is shared among the siblings and cousins of *t *; a label *l_t _*which comes from the category of the parent of *t *and which is not shared with any of the siblings of *t*; and a multi-label Bayesian classifier *B_Ct,St _*using definition *1*. The training set *St *has the following restriction: *∀s ∈ S_t _, L_t∗ _⊆ label(s)*. Finally, we require that the label of the root node be empty.

**Definition 10**. If *B_t _*is the Bayesian classifier associated with node *t *and *I *is an object which maybe categorized by *B_t_*, then *B_t_(I) *is the list of all labels which *B_t _*returns upon classifying *I*.

**Definition 11**. If *l *is a label and *t *is a node in a tree then *Child(l, t) *is the child of *t*, which contains label *l*.

Building the Bayesian decision tree

**Figure 3:**
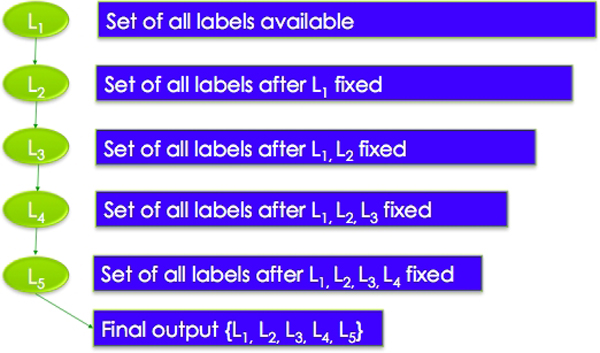
**Less is More**. The Bayesian Decision Tree limits the number of labels at each node. The pruning is done on the basis of the F1 micro score from the gold standard annotations. Thus the naïve Bayes process can be applied to a more concentrated set of abstract-label combinations resulting in more accurate annotations.

Using these definitions, we construct a framework for annotating the neuroimaging abstracts with labels from the CogPO ontology categories of SM, ST, RM, RT, and I. We limit the training set on the naïve Bayes classifiers in the tree in order to leverage the dependencies that exist between labels in different categories. Thus we change the underlying probabilities of the Bayesian classifier to better fit any dependencies between labels in different categories. This *less is more *approach helps the Bayesian classifier to focus on attributes that are more important to the current node, as seen in Figure 3.

Our approach uses conditional learning to boost accuracy and recall in automatic learning systems. By conditional learning we mean that when the system classifies an abstract, it uses stochastic models (naive Bayes classifier's in this case) that were built with training data that is limited to only those training items that have labels that were already determined to be pertinent to the abstract currently being labeled on a higher level of the decision tree Table 2.

**Table 2:** High level description of the algorithm.

**Input** • Un-Labeled Item I • Bayesian Decision Tree T **Output** • Label Vector in Multiple Categories L **Algorithm** t = Root(T) SearchList = NULL **while **t ~= NULL **do** L = L : Bt(I) **for **l ϵ B_t_(I) **do** SearchList = SearchList : Child(l, t) **end for** t = SearchList[0] x : SearchList = SearchList **end while** return L

This recursive program uses the Bayesian Decision Tree defined in Definition 9, along with Bayesian Classifier of Definition 10 and the child function of Definition 11 to label an unlabeled item. Unlike a normal naive Bayes classifier that is trained on the whole training set, this algorithm steps through a decision tree whose every node contains a classifier that is trained on a narrow subset of the original training set. This subset is limited to only those items whic h are annotated with the labels of the ancestors of the current node.

For example, consider an abstract that is being evaluated by this system and that has already been tagged by the system as having a *Stimulus Modality *of *Auditory*. When the system reaches the *Stimulus Type *level of the decision tree, it will reach for a naive bayes classifier that has *not *been trained on the entire gold-standard data set. Instead it will reach for a classifier which has been trained only on abstracts that were known to have *Auditory *as a label. This means that the underlying probabilities of various labels for *Stimulus Type *will change, making a label like *Chord Sequences*, a inherent *Stimulus Type *of *Auditory *more likely, and making a *Stimulus Type *of *False Fonts*, from *Stimulus Modality Visual*, less likely.

It is important to note that this is not because the algorithm has been programmed to explicitly avoid the *Stimulus Type *label *False Fonts *when it encounters an abstract already labeled *Auditory*. Instead this is due to the fact that it is implicitly the case in the literature and given CogPO that the *False Fonts *label is mostly not compatible with the *Auditory *label, and human annotators, with their natural understanding of both the meaning of the literature and the ontology, capture this fact in their annotations. Our process merely retrieves this underlying implicit understanding from the annotations in the literature and then leverages that structure to aid in the annotation process.

We asses the performance of our approach using the F1-micro score, based on precision and recall [28]. In all our calculations, we set β=1

$$F_{\beta }=\left(1+\beta^{2}\right)\frac{precision*recall}{\beta^{2}*precision+recall}$$

We first construct 5 separate naïve Bayes classifiers for each of the 5 categories as formalized in section 2.2. Each classifier is then trained and tested on the entire corpus of abstracts using 10-fold cross-validation, and their F1-micro scores are calculated. Abstracts in the testing set are annotated with a label if the label had a probability score greater than *F_β _= 0.1*.

Next we construct the Bayesian Decision Trees as formalized in the previous section. Given that we have 5 categories, we build all 120 possible BDTs. We annotate the corpus of abstracts using the BDTs with the criterion that if the probability of a label is greater than *0.1 *for some abstract, then that abstract is tagged with that label. Next we aggregate the labels across each of the 5 categories and calculate a mean F-score for each category to determine the quality of the annotations for each instance of the category across all trees as seen in Figure 3.

Our approach can also be extended to the case in which the human subject matter expert is in the classification loop and has an input to the automated annotation process, that is, the human subject matter expert is using our algorithm to more efficiently annotate the set of abstracts. A human subject matter expert can usually determine the label for at least one of the categories with a quick glance at an abstract. For exmaple, if the abstract explicitly states that the experiment used a picture of faces as the stimulus, or that subjects pushed a button with their foot to respond. To model this, we trained our BDTs with the condition that the root node has already been decided. We call this the Constrained Decision Tree (CDT). As a result we have trees rooted at *SM, ST, RM, RT*, and *I*, corresponding to the cases where the human expert assigns the label for that category. The rest of the tree is constructed exactly as before except that, when the mean f-score is calculated for each category across all possible CDTs, we remove the instances corresponding to the annotations assigned by the human subject matter expert since we do not want them to influence the results returned by our algorithm.

## Results and discussions

**Figure 4:**
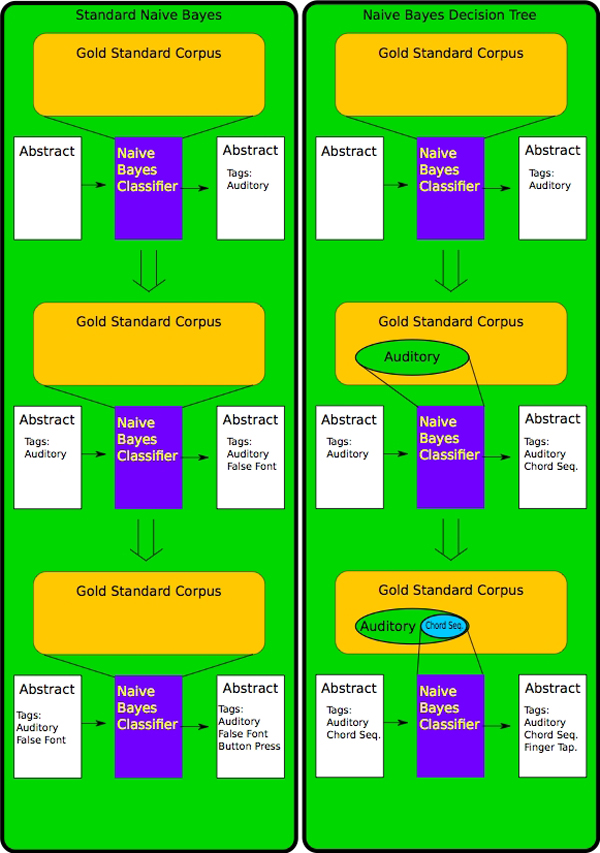
**Decision Trees**. In this figure we can see an abstract going through a few steps of the annotation process for both a regular naive Bayes classifier trained on the gold standard corpus and a Bayesian decision tree. The abstract classified by the naive Bayes classifier is classified without regard to decisions already made by the classifier. Therefore, it is classified with the label *False Font *as its stimulus modality even though its stimulus type was *Auditory*. By contrast, the when the Bayesian decision tree needs to identify a *Stimulus Type *it uses a classifier trained on a set of abstracts which are all annotated with the label *Auditory *and thus picks *Chord Sequence *as the abstract's *Stimulus Type*.

Figure 4. shows an overview of the entire process. The first task of the annotation process is handled by the naïve Bayes algorithm. The output of the naïve Bayes algorithm is then used by the Bayesian decision tree algorithm to calculate the annotation tags.

**Figure 5:**
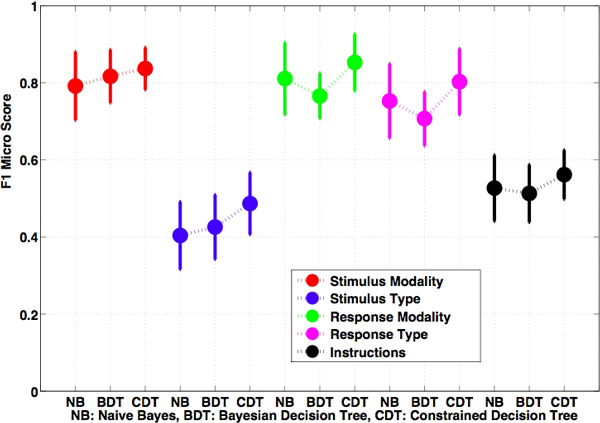
**Comparison of Methods**. F1 micro scores for the annotation returned for the Stimulus Modality, Stimulus Type, Response Modality, Response Type, and Instructions. The error bars are twice the standard deviation.

Our results are shown in Figure 5. The error bars presented are twice the standard deviation with respect to the mean of the F1-micro score for each category. F1-micro scores for *Stimulus Type *(ST) and *Instructions *(I) are lower than in the other categories because of the large number of labels they incorporate, leading to lower relative sample size for each label. *Stimulus Modality *(SM), *Response Modality *(RM), and *Response Type *(RT) have fewer labels and thus produce better performance.

For *Response Modality *(RM), *Response Type *(RT), and *Instructions *(I), the Decision Tree F1-micro score is slightly lower than that of the naïve Bayes because our sample size constriction for the training sets at each level of the decision tree decreases precision and recall for labels lower down in the tree, and any increases due to underlying correlations are not sufficient to make up for this decrease. The Constrained Decision Tree always has a higher F1-micro score than the other methods because the guarantee of correct labels in the first category of each tree is leveraged through the cascading correlations among labels in different categories further down the tree and the labels discovered in the root node's category.

The combination of the stochastic representational power of the naïve Bayes with the expressive simplicity of the Bayesian Decision Trees allows our automated classifier to achieve a significant improvement in the annotation of literature as compared to existing string-matching tools like the NCBO Annotator. Not only are we able to annotate across multiple categories, but our method also captures the implicit structural dependencies induced in the set of labels found in the gold standard labelled corpus. Of course, this capture process will vary with the corpus to which it is applied, and a different corpus for the same ontology being modeled by the same gold standard will produce a different reification of the dependencies captured in the form of annotations across categories. Thus, instead of explicitly modeling the relationships between superclasses and classes directly from the ontology, we have developed a stochastic model that can capture the effect of those superclass-class relationship indirectly from the specific combination of human annotations and the corpus. Thus the same stochastic meta-algorithm can be applied to solve similar automated annotation problems with different ontologies, as well as a different gold standard for that ontology applied to several different corpora.

The constrained human-in-the-loop decision tree architecture further improves upon the naïve Bayes results. When we fix the first node of the decision tree, there is a significant improvement in the annotation accuracy. This is a useful tool for aiding a human expert in annotation because the expert can usually select one annotation from several categories with a quick skim of an abstract. Our technique can then annotate the remaining categories with high accuracy. Although this approach does not eliminate the human expert from the loop, it complements their decision-making and has the potential to reduce the time and effort for the full annotation task.

## Conclusions and future work

We have demonstrated a stochastic framework for annotating BrainMap literature using the Cognitive Paradigm Ontology. Unlike text mining algorithms, our framework can model the knowledge encoded by the dependencies in the ontology, albeit indirectly. We successfully exploit the fact that CogPO has explicitly stated restrictions, and implicit dependencies in the form of patterns in the expert curated annotations. The advantage of our pragmatic approach is that it is robust to explicit future modifications and additions that could be made to the relationships and restrictions in CogPO. Since we do not explicitly model the relations and restrictions, but capture them implicitly from training patterns, we do not have to make corresponding updates to our algorithm each time CogPO is updated by humans. We merely need to have a correctly annotated body of work.

The constrained decision tree architecture further improves upon the naïve Bayes results. When we fix the first node of the decision tree, there is a significant improvement in the annotation accuracy. This is a useful tool for aiding a human expert in the annotation task.

We next plan to apply our techniques to different ontologies with more complex structures. We believe the modular nature of our framework will scale well to these new ontologies. There is additional progress to be made in algorithmically learning gaps (missing labels) in the ontology. We speculate that our technique can find missing restrictions and relations not explicitly defined in CogPO.

## Competing interests

The authors declare that they have no competing interests.

## Authors' contributions

CC and TBJ designed and implemented the experiments, the algorithms, and the formal framework. GFL and JAT are the PI's of the project and secured the funding. GFL, as the computer science lead, coordinated the technical aspects of the research. GFL and JAT supervised the development tasks of the project. MDT performed statistical testing and analysis. ARL created the gold standard corpus. JFW implemented helper functions and other utilities.

## Declarations

Ths article is published as part of a supplement Bio-Ontologies 2013. This supplement is supported by NIMH / NIH awards R56-MH097870, R01-MH084812, and R01-MH074457 and by the Department of Computer Science of the University of New Mexico.

This article has been published as part of *Journal of Biomedical Semantics *Volume 5 Supplement 1, 2014: Proceedings of the Bio-Ontologies Special Interest Group 2013. The full contents of the supplement are available online at http://www.jbiomedsem.com/supplements/5/S1.
